# Cocaine, nicotine, and their conditioned contexts enhance consolidation of object memory in rats

**DOI:** 10.1101/lm.048579.118

**Published:** 2019-02

**Authors:** Michael Wolter, Ethan Huff, Talia Speigel, Boyer D. Winters, Francesco Leri

**Affiliations:** Department of Psychology and Collaborative Program in Neuroscience, University of Guelph, Guelph, Ontario N1G 2W1, Canada

## Abstract

To test the hypothesis that drugs of abuse and their conditioned stimuli (CSs) enhance memory consolidation, the effects of post-training exposure to cocaine and nicotine were compared to the effects of post-training exposure to contextual stimuli that were paired with the effects of these drugs. Using the object recognition (OR) task, it was first demonstrated that both 10 and 20 mg/kg cocaine, and 0.2 and 0.4 mg/kg nicotine, enhanced recognition memory when administered immediately after, but not 6 h after the sample phase. To establish the drug CSs, rats were confined for 2 h in a chamber (the CS+) after injections of 20 mg/kg cocaine, or 0.4 mg/kg nicotine, and in another chamber (the CS−) after injections of vehicle. This was repeated over 10 d (5 drug/CS+ and 5 vehicle/CS− pairings in total). At the end of this conditioning period, when tested in a drug-free state, rats displayed conditioned hyperactivity in the CS+ relative to the CS−. More important, immediate, but not delayed, post-sample exposure to the cocaine CS+, or nicotine CS+, enhanced OR memory. Therefore, this study reports for the first time that contextual stimuli paired with cocaine and nicotine, like the drugs themselves, have the ability to enhance memory consolidation.

Thorndike (1911) proposed that a reinforcer acts as an event that “stamps-in” the association between stimuli and responses. This idea has been formalized by the hypothesis that reinforcers exert their behavioral effects by enhancing memory consolidation: a time-dependent process in which a memory trace becomes stabilized and less sensitive to interference (White and Milner 1992; McGaugh and Roozendaal 2009). Biologically, this is significant because events that enhance memory consolidation also increase the probability that behaviors will be more likely to be repeated in the future (White 1996).

The experimental approach used to explore the memory enhancing function of reinforcers involves manipulations delivered immediately, or soon after, training on a given task (White 1996; Rkieh et al. 2014). This is a key experimental requirement because it is believed that a memory trace is labile, and therefore sensitive to modulations, particularly during a critical period of minutes to hours that follow the experience of learning (McGaugh 2000). Therefore, using this post-training approach, it has been demonstrated that reinforcers such as food (Huston et al. 1974, 1977), sucrose (Messier and White 1984), and various drugs of abuse (Introini-Collison and McGaugh 1989; Janak et al. 1992; Blaiss and Janak 2006; Iñiguez et al. 2012; Leri et al. 2013) improve learning of a variety of tasks in several species (Eddins et al. 2009; Iñiguez et al. 2012; May et al. 2016).

An interesting question is whether cues paired with reinforcing stimuli via classical conditioning can also influence memory consolidation. These are usually referred to as conditioned stimuli (CS), or conditioned reinforcers, depending on the behavioral effect of interest. For example, activation of drug-paired CSs enhance operant responses in the absence of drugs (Di Ciano and Everitt 2003), and can even maintain responding when delivered contingently (Rescorla and Solomon 1975; Tunstall and Kearns 2017). Moreover, in place conditioning, when a drug reinforcer is administered in a specific context, the contextual CS gains the ability to attract the animal when in a drug-free state (for review, see Tzschentke 1998), and can induce ultrasonic vocalizations similarly to acute injections of the drug (Ahrens et al. 2009; Ma et al. 2010; Hamed et al. 2012). Drug-paired CSs also acquire the ability to elicit other behavioral responses (e.g., conditioned locomotion) and modify various physiological functions (e.g., heart and respiratory rates) (Bloch et al. 1973; Fitzgerald et al. 1984; Blanco et al. 2012).

While it has been repeatedly demonstrated that CSs established by conditioning with drugs of abuse can activate and maintain approach behavior, it is unclear whether these CSs can also modulate memory consolidation. Holahan and White (2004) found that post-training exposure to a fear provoking CS enhanced consolidation of a cue preference task. As well, Leong et al. (2015) and Goode et al. (2016) reported that the impact of these CSs can be selective to particular memory systems and that the pharmacological inhibition of conditioned arousal blocks their impact on learning. However, Holahan and White (2013) demonstrated that post-training exposure to a sucrose-paired context was also effective in enhancing cue preference learning, suggesting that conditioned fear/arousal is not always necessary to enhance memory consolidation. This conclusion is further supported by evidence of enhanced acquisition of the Morris water maze by pretesting exposure to a morphine- or cocaine-paired context (Zhai et al. 2007), although this result cannot confirm that the drug-paired appetitive context impacted memory consolidation because pretraining manipulations preclude conclusions about the selective stage of learning affected (encoding vs. consolidation).

The experiments presented in the current study were designed to test the hypothesis that drug CSs can enhance memory consolidation by comparing the effects of post-training drug administration to the effects of post-training exposure to contextual stimuli that were paired with the effects of the same drugs. Cocaine and nicotine were selected because they have been found previously to enhance memory consolidation (Introini-Collison and McGaugh 1989; Beer et al. 2013) and because they support classical conditioning of various responses (Jackson et al. 2009; Johnson et al. 2012). Contextual conditioning was selected because place preference studies performed in several laboratories, including ours, have consistently shown that a compartment paired with injections of cocaine will elicit an approach response in cocaine-free animals (Bardo et al. 1995; Calcagnetti et al. 1995; Leri et al. 2006; Sticht et al. 2010; Thériault et al. 2018). Place preference studies with nicotine point to the same general conclusion (Le Foll and Goldberg 2005, 2009), although the results with nicotine have been more variable (Liu et al. 2008), and it appears that the exact conditioning parameters are very important to nicotine contextual conditioning (Risinger and Oakes 1995; Tzschentke 1998; Le Foll and Goldberg 2005).

The effects of post-training administration of cocaine and nicotine, and exposure to cocaine and nicotine CSs, were tested on a spontaneous object recognition (OR) task. This memory task is based on the natural tendency of rats to explore novel objects (Ennaceur and Meliani 1988; Winters et al. 2004), and it was selected because of our previous demonstration that OR 72 h after learning can be improved by post-sample administration of cocaine (Rkieh et al. 2014).

A nicotine place conditioning experiment was also included to verify whether the CS+ established in our apparatus and with the selected conditioning protocol would be effective in revealing conditioned approach, another key aspect of reinforcement (White and Milner 1992). A cocaine place conditioning experiment was not deemed necessary because we have found that rats will consistently approach a compartment of our conditioning apparatus paired with 20 mg/kg cocaine (Leri et al. 2006; Sticht et al. 2010; Cummins Jacklin et al. 2015; Thériault et al. 2018).

## Results

### Experiment 1

**Figure 1.**
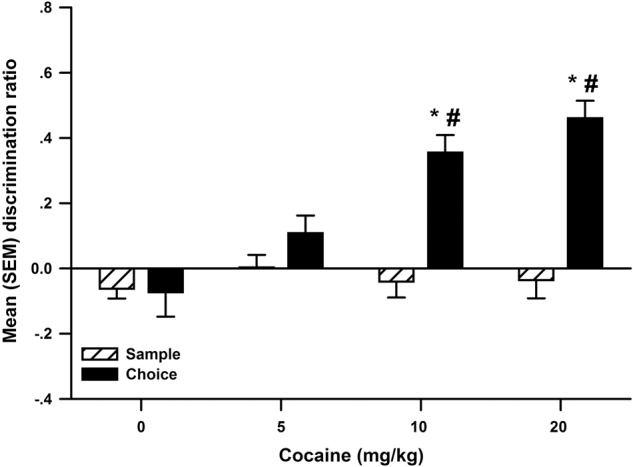
The mean (±SEM) discrimination ratio from the sample and choice phases of object recognition following post-sample injections of 0 (*n* = 23), 5 (*n* = 23), 10 (*n* = 23), and 20 (*n* = 23) mg/kg cocaine in Experiment 1. The * denotes a significant difference compared to 0 mg/kg cocaine choice phase discrimination ratio. The # denotes a significant difference when compared to the sample phase discrimination ratio.

Immediate post-sample cocaine enhanced OR memory (Fig. 1). The ANOVA revealed a significant interaction between Dose and Phase (*F*_(3,66)_ = 10.73, *P* < 0.001), as well as significant main effects of Dose (*F*_(3,66)_ = 6.61, *P* < 0.001) and Phase (*F*_(1,66)_ = 57.09, *P* < 0.001). Multiple comparisons further indicated that, when rats were injected with 10 and 20 mg/kg cocaine, their choice discrimination ratio was significantly higher compared to their sample discrimination ratio, as well as 0 mg/kg cocaine choice discrimination ratio. The analysis of total object exploration was nonsignificant for the sample and choice phases (data not shown). When cocaine injections were delayed by 6 h, there was no evidence of object memory, as the sample and choice phase discrimination ratios did not differ (Fig. 5A).

### Experiment 2

**Figure 2.**
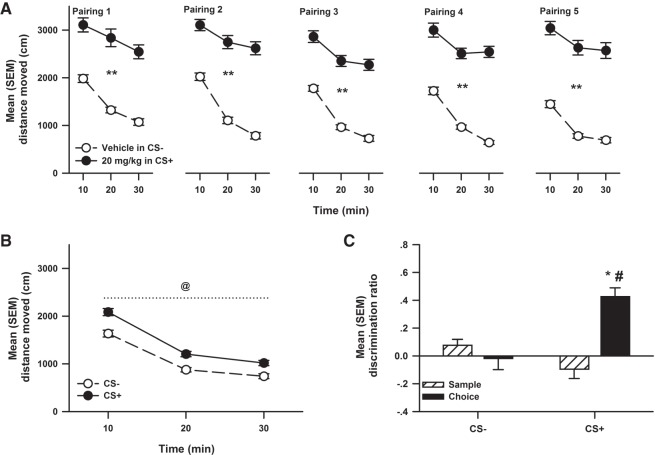
Experiment 2. (*A*) Mean (±SEM) distance moved in compartments paired (1–5) with injections of Vehicle (in CS−; *n* = 48) and 20 (in CS+; *n* = 48) mg/kg cocaine. The ** denotes a significant difference compared to CS− distance moved at all time points. (*B*) The mean (±SEM) distance moved during the 30 min test of conditioned locomotion in the compartment previously paired with Vehicle (CS−) and 20 mg/kg (CS+) (*n* = 48) cocaine. The @ denotes a significant difference compared to CS− distance moved. (*C*) The mean (±SEM) discrimination ratio produced during the sample and choice phase of object recognition following exposure to CS compartments previously paired with Vehicle (CS−) and 20 mg/kg (CS+) (*n* = 12) cocaine post-sample. The * denotes a significant difference compared to CS− choice phase discrimination ratio. The # denotes a significant difference compared to sample phase discrimination ratio.

Significant conditioned locomotion was observed in the compartment paired with cocaine, and immediate post-sample exposure to this CS+ enhanced object memory (Fig. 2). Figure 2A represents the mean (±SEM) distance moved in compartments paired (1–5) with injections of 0 (in CS−) and 20 (in CS+) mg/kg cocaine. The ANOVA revealed significant interactions between Dose and Time (*F*_(2,705)_ = 16.48, *P* < 0.001), Dose and Pairing (*F*_(4,705)_ = 4.69, *P* = 0.001), as well as significant main effects of Dose (*F*_(1,705)_ = 1748.87, *P* < 0.001), Time (*F*_(2,705)_ = 119.61, *P* < 0.001), and Pairing (*F*_(4,705)_ = 8.88, *P* < 0.001). Multiple comparisons further indicated that when rats were injected with 20 mg/kg cocaine in the CS+, they were significantly more active than when they were injected with 0 mg/kg cocaine in the CS− at each time point and pairing.

Figure 2B represents the mean (±SEM) distance moved during the test of conditioned locomotion (30 min) in compartments previously paired with 0 (CS−) and 20 mg/kg (CS+) cocaine. The ANOVA only revealed significant main effects of CS compartment (*F*_(1,94)_ = 49.25, *P* < 0.001) and Time (*F*_(2,94)_ = 344.82, *P* < 0.001). Multiple comparisons of marginal means further indicated that rats displayed significantly higher locomotor activity in the CS+ than in the CS− compartments.

Figure 2C represents mean (±SEM) discrimination ratio produced during the sample and choice phase of OR following exposure to CS compartments previously paired with 0 (CS−) and 20 mg/kg (CS+) cocaine post-sample. The ANOVA revealed a significant interaction between Compartment and Test (*F*_(1,11)_ = 21.40, *P* < 0.001), as well as significant main effects of Compartment (*F*_(1,11)_ = 5.99, *P* = 0.03) and Test (*F*_(1,11)_ = 26.55, *P* < 0.001). Multiple comparisons further indicated that when rats were exposed to the CS+ compartment post-sample, their choice discrimination ratio was significantly higher when compared to their sample discrimination ratio and CS− choice discrimination ratio. The analysis of total object exploration was nonsignificant for the sample and choice phases (data not shown). When exposure to the CS compartments previously paired with 0 (CS−) and 20 mg/kg (CS+) cocaine was delayed by 6 h, there was no evidence of object memory (Fig. 5B), as the discrimination ratios did not differ between sample and choice phases.

A final analysis ascertained whether the choice DR following post-training confinement in the cocaine-paired compartment was related to total locomotion displayed during the confinement period, and the correlation was not statistically significant.

### Experiment 3

**Figure 3.**
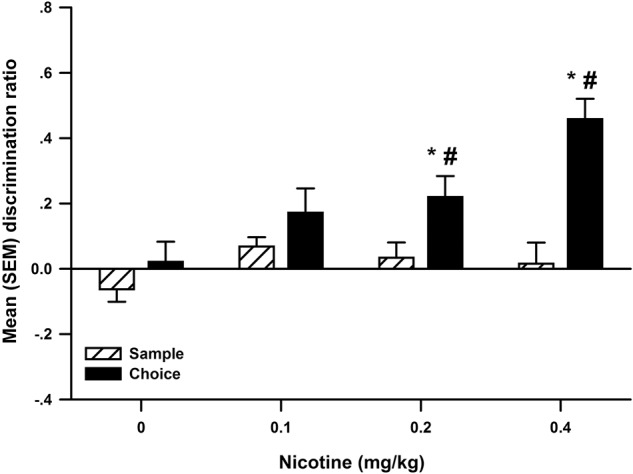
The mean (±SEM) discrimination ratio from the sample and choice phases of object recognition following post-sample injections of 0 (*n* = 23), 0.1 (*n* = 23), 0.2 (*n* = 23), and 0.4 (*n* = 23) mg/kg nicotine in Experiment 3. The * denotes a significant difference compared to 0 mg/kg nicotine choice phase discrimination ratio. The # denotes a significant difference compared to sample discrimination ratio.

Immediate post-sample nicotine enhanced OR performance (Fig. 3). The ANOVA revealed a significant interaction between Dose and Phase (*F*_(3,66)_ = 4.70, *P* = 0.005), as well as significant main effects of Dose (*F*_(3,66)_ = 9.63, *P* < 0.001) and Phase (*F*_(1,66)_ = 20.67, *P* < 0.001). Multiple comparisons further indicated that rats only produced significantly higher discrimination ratios when injected with 0.2 and 0.4 mg/kg nicotine when compared to the sample discrimination ratio and 0 mg/kg nicotine choice discrimination ratio. The analysis of total object exploration was nonsignificant for the sample and choice phase (data not shown). When nicotine (0.4 mg/kg) was delayed by 6 h, there was no difference between the sample and choice discrimination ratios (Fig. 5C).

### Experiment 4

**Figure 4.**
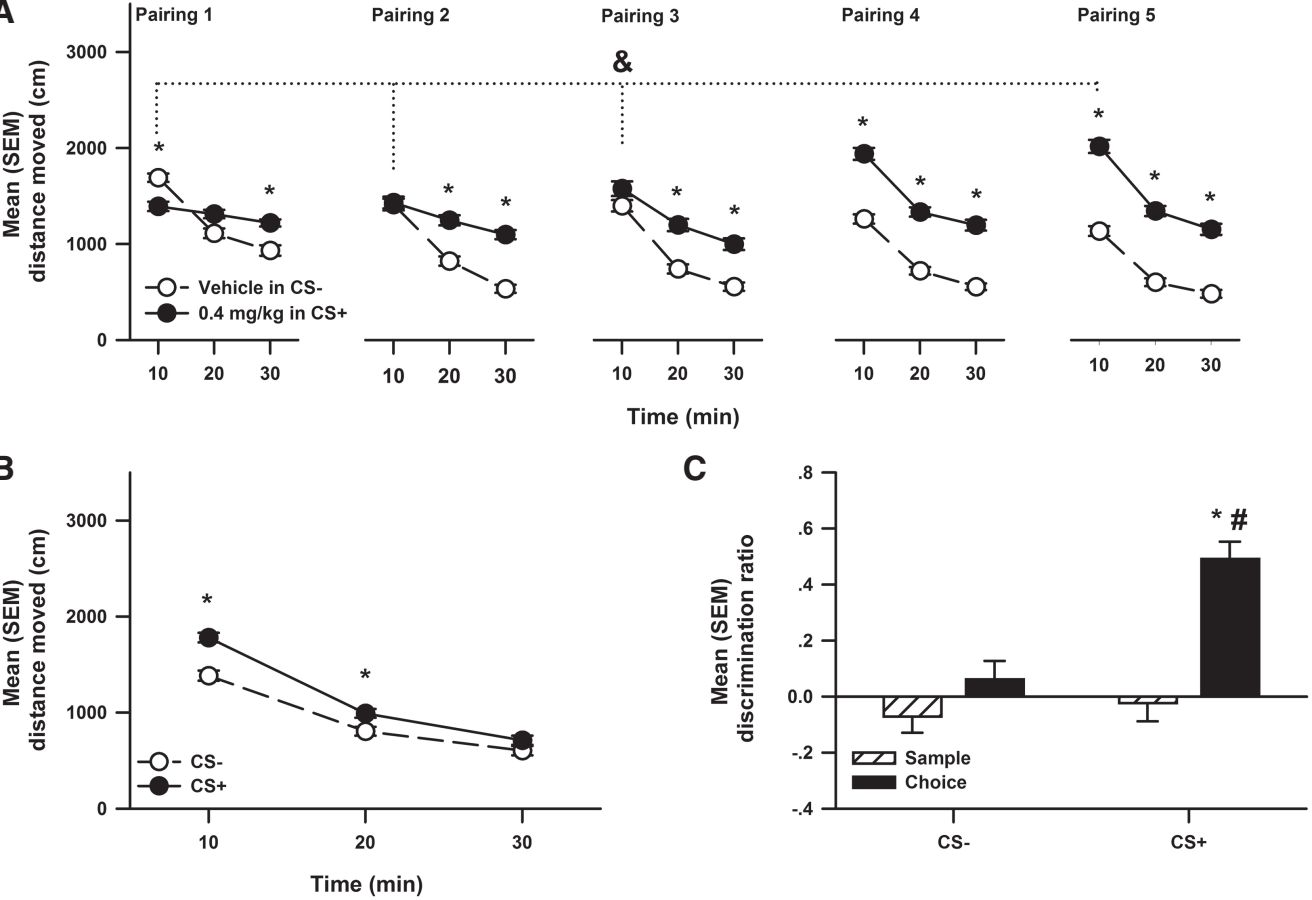
Experiment 4. (*A*) Mean (±SEM) distance moved during pairings (1–5) of locomotion to compartments paired with injections of vehicle (in CS−; *n* = 48) and 0.4 (in CS+; *n* = 48) mg/kg nicotine after 30 min. The * denotes a significant difference compared to Vehicle CS−. The & denotes a significant difference compared to 0.4 mg/kg in CS+ nicotine pairing 5. (*B*) The mean (±SEM) distance moved during the 30 min test of conditioned locomotion in compartments previously paired with Vehicle (*n* = 48) (CS−) and 0.4 (*n* = 48) mg/kg (CS+) nicotine. The * denotes a significant difference compared to CS− distance moved. (*C*) The mean (±SEM) discrimination ratio calculated during the sample and choice phases of object recognition following exposure to a compartment previously paired with Vehicle (*n* = 12) (CS−) and 0.4 (*n* = 12) mg/kg (CS+) nicotine post-sample. The * denotes a significant difference compared to CS− choice phase discrimination ratio. The # denotes a significant difference compared to sample discrimination ratio.

Significant conditioned locomotion was observed in the compartment paired with nicotine, and immediate post-sample exposure to this CS+ enhanced object memory (Fig. 4). Figure 4A represents the mean (±SEM) distance moved in compartments paired (1–5) with injections of 0 (in CS−) and 0.4 (in CS+) mg/kg nicotine. The ANOVA revealed significant interactions between Dose, Time, and Pairing (*F*_(8,564)_ = 10.25, *P* < 0.001), Dose and Time (*F*_(4,564)_ = 4.93, *P* = 0.03), Dose and Pairing (*F*_(4,564)_ = 81.82, *P* < 0.001), as well as Time and Pairing (*F*_(8,564)_ = 7.56, *P* < 0.001). The analysis also revealed significant main effects of Dose (*F*_(1,54)_ = 40.29, *P* < 0.001), Time (*F*_(2,564)_ = 106.52, *P* < 0.001), and Pairing (*F*_(4,564)_ = 24.99, *P* < 0.001). Multiple comparisons indicated that rats were significantly more active when injected with 0.4 mg/kg nicotine in the CS+ during pairings 4 and 5 compared to pairings 1 and 2 and when they were injected with 0 mg/kg nicotine in the CS−.

Figure 4B represents the mean (±SEM) distance moved during the 30 min test of conditioned locomotion in the compartment previously paired with 0 (CS−) and 0.4 mg/kg (CS+) nicotine. The ANOVA revealed a significant interaction between CS compartment and Time (*F*_(2,94)_ = 10.12, *P* < 0.001), as well as significant main effects of CS compartment (*F*_(1,94)_ = 30.86, *P* < 0.001) and Time (*F*_(2,94)_ = 438.77, *P* < 0.001). Multiple comparisons further indicated that rats placed into the CS+ compartment were significantly more active than rats placed into the CS− compartment at 10 and 20 min.

Figure 4C represents the mean (±SEM) discrimination ratio calculated during the sample and choice phases of OR following exposure to a compartment previously paired with 0 (CS−) and 0.4 mg/kg (CS+) nicotine post-sample. The ANOVA revealed a significant interaction between CS compartment and Phase (*F*_(1,9)_ = 11.824, *P* = 0.007), as well as significant main effects of CS compartment (*F*_(1,9)_ = 15.27, *P* = 0.004) and Phase (*F*_(1,9)_ = 23.10, *P* < 0.001). Multiple comparisons further indicated that when rats were exposed to the CS+ compartment post-sample, their choice discrimination ratio was significantly higher when compared to their sample discrimination ratio and CS− choice discrimination ratio. The analysis of total object exploration was nonsignificant for the sample and choice phases (data not shown). A final analysis ascertained whether choice DR following post-training confinement in the cocaine-paired compartment was related to total locomotion displayed during the confinement period, and the correlation was not statistically significant.

**Figure 5.**
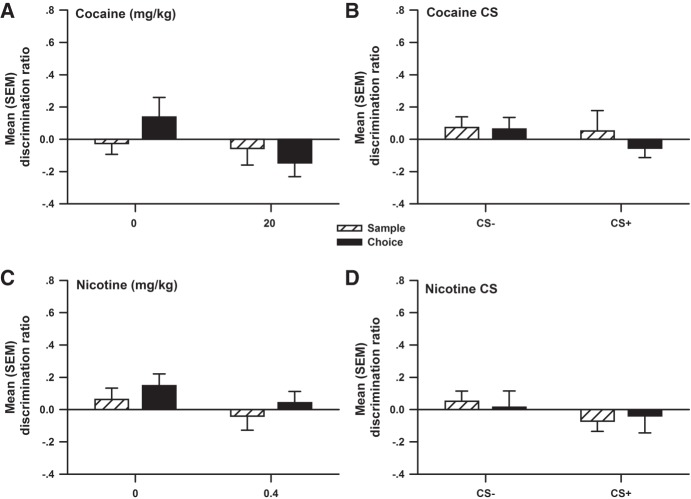
Experiment 5. (*A*) The mean (±SEM) discrimination ratio produced during the sample and choice phases of object recognition in response to an injection of 0 (*n* = 12) and 20 (*n* = 12) mg/kg cocaine 6 h post-sample. (*B*) The mean (±SEM) discrimination ratio produced during the sample and choice phases of object recognition in response to exposure to a compartment previously paired with 0 (*n* = 12) (CS−) and 20 mg/kg (*n* = 12) (CS+) cocaine 6 h post-sample. (*C*) The mean (±SEM) discrimination ratio produced during the sample and choice phase of object recognition in response to an injection of 0 (*n* = 12) and 0.4 (*n* = 12) mg/kg nicotine 6 h post-sample. (*D*) The mean (±SEM) discrimination ratio produced during the sample and choice phase of object recognition in response to exposure to a compartment previously paired with 0 (*n* = 12) (CS−) and 0.4 (*n* = 12) mg/kg (CS+) nicotine 6 h post-sample. There was no evidence of object recognition in any condition when the drug or CS+ exposure was delay by 6 h.

When exposure to the CS compartments previously paired with 0 (CS−) and 0.4 mg/kg (CS+) nicotine was delayed by 6 h the discrimination ratios in the sample and choice phases did not differ (Fig. 5D).

### Experiment 5

**Figure 6.**
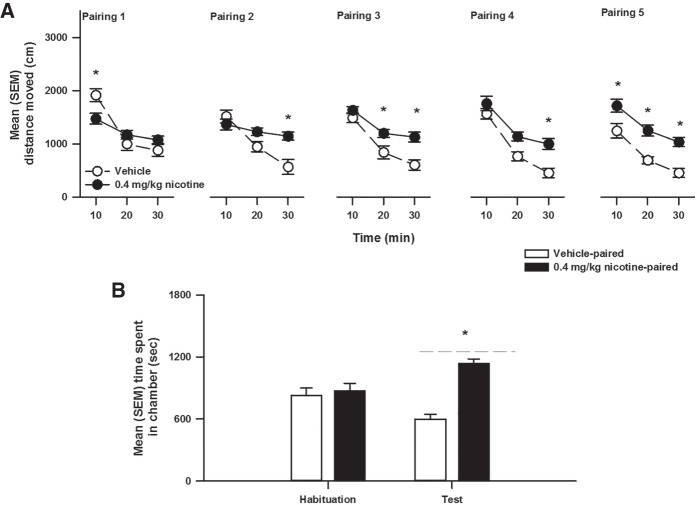
(*A*) Mean (±SEM) distance moved during pairings (1–5) of locomotion to compartments paired with injections of vehicle (*n* = 11) and 0.4 (*n* = 11) mg/kg nicotine after 30 min. The * denotes a significant difference compared to 0 mg/kg in CS−. (*B*) The mean (±SEM) time spent in a vehicle-paired (*n* = 11) and 0.4 mg/kg nicotine-paired (*n* = 11) chamber during the habituation and test of conditioned place preference. The * denotes a significant difference compared to the vehicle-paired chamber.

During the test of conditioned place preference, rats significantly preferred the nicotine-paired chamber over the vehicle-paired chamber (Fig. 6). Figure 6A represents the mean (±SEM) distance moved in compartments paired (1–5) with injections of vehicle and 0.4 mg/kg nicotine. The ANOVA revealed significant interactions between Dose and Time (*F*_(2,329)_ = 23.82, *P* < 0.001), Dose and Pairing (*F*_(4,329)_ = 6.98, *P* = 0.002); however, the interaction between Time and Pairing as well as Drug, Time, and Pairing were nonsignificant. The analysis also revealed significant main effects of Dose (*F*_(1,329)_ = 57.62, *P* < 0.001) and Time (*F*_(2,329)_ = 193.27, *P* < 0.001), but not Pairing. Multiple comparisons indicated that rats were significantly more active when injected with 0.4 mg/kg nicotine in the nicotine-paired chamber than when they were injected with vehicle in the vehicle-paired chamber.

Figure 6B represents the mean (±SEM) time spent in the chambers paired with vehicle and nicotine (0.4 mg/kg). The ANOVA revealed a significant interaction between Chamber and Phase (*F*_(1,43)_ = 15.57, *P* = 0.003), as well as a significant main effect of Chamber (*F*_(1,43)_ = 7.03, *P* = 0.02), but not Phase. Multiple comparisons indicated that during habituation rats did not significantly prefer either chamber but spent significantly more time in the nicotine-paired chamber during the test of place preference.

## Discussion

To test the hypothesis that incentive CSs enhance memory consolidation, this study compared the effects of post-training exposure to cocaine, nicotine, and contextual stimuli paired with the effects of these drugs on object memory in rats. Using the OR task, it was first demonstrated that both 10 and 20 mg/kg cocaine, and 0.2 and 0.4 mg/kg nicotine, can enhance recognition memory when administered immediately, but not 6 h, after the sample phase of OR. To establish the drug contextual CSs, rats were confined for 2 h in a chamber (the CS+) after injections of 20 mg/kg cocaine, or 0.4 mg/kg nicotine, and in another chamber (the CS−) after injections of vehicle. At the end of conditioning, when tested in a drug-free state, animals displayed conditioned hyperactivity in the CS+ relative to the CS−. More important, immediate but not delayed exposure to the cocaine CS+, or to the nicotine CS+, enhanced recognition memory. Therefore, this study reports for the first time that contextual stimuli paired with drugs of abuse not only gain the ability to produce approach, but they also become capable of enhancing memory processes.

Cocaine alters synaptic levels of dopamine, noradrenaline, and serotonin by blocking their transporters (Carrera et al. 2004). Nicotine activates nicotinic acetylcholine receptors throughout the brain (Deiana et al. 2011), also enhancing levels of monoamine neurotransmitters (Berrettini 2008). Both drugs are abused (Carrera et al. 2004; Le Foll and Goldberg 2006), and both should enhance memory consolidation when administered post-training, as predicted by the hypothesis of White and Milner (1992). This prediction has been tested extensively in several species using various memory tasks (Introini-Collison and McGaugh 1989; Sansone et al. 1991; Puglisi-Allegra et al. 1994; Ciamei et al. 2000, 2001; Sharifzadeh et al. 2005; Iñiguez et al. 2012), and in general, the results are consistent with this prediction. Using the OR task, the current study sought to expand these findings to OR memory by immediate or delayed post-sample administration of cocaine (0, 5, 10, 20 mg/kg) and nicotine (0, 0.1, 0.2, 0.4 mg/kg). As expected, both cocaine (Fig. 1) and nicotine (Fig. 3) produced dose-dependent increases in recognition memory, replicating the findings of Rkieh et al. (2014) for cocaine and of Puma et al. (1999) for nicotine. Importantly, Experiment 5 revealed no significant difference when the injections of 20 mg/kg cocaine (Fig. 5A) or 0.4 mg/kg nicotine (Fig. 5C) were delayed by 6 h following the sample phase. This strongly suggests that post-training administration of these drugs enhanced memory of the objects seen during the sample phase because of a selective action on consolidation rather than other memory processes such as encoding or retrieval (Roozendaal and McGaugh 2012).

Demonstrating that both post-sample cocaine and nicotine effectively enhanced OR memory was essential to test whether contextual stimuli paired with these drugs could also modify memory consolidation in the same task. Hence, using a place conditioning protocol, rats received 20 mg/kg cocaine (Experiment 2), 0.4 mg/kg nicotine (Experiment 4), or their vehicle, prior to confinement to two distinct conditioning chambers (the CS+ and the CS−), respectively. During these pairings, the typical stimulation of motor activity was observed (Figs. 2A, 4A, and 6A). Importantly, when locomotion was tested in a drug-free state, animals conditioned with cocaine or nicotine moved significantly more in the CS+ than in the CS− (Figs. 2B and 4B), clearly indicating that the CS+ had acquired the ability to produce an observable conditioned response on motor behavior (Johnson et al. 2012). More important, using this within-subjects design, it was found that post-sample exposure to the cocaine (Fig. 2C) or to the nicotine (Fig. 4C) CS+ significantly enhanced object memory in comparison to when animals were exposed to the CS−.

This primary finding most likely reflects an enhancement of consolidation by exposure to the CS+, rather than an inhibition caused by exposure to the CS−, as the 72 h version of OR used in this study can only reveal memory facilitation. Furthermore, it is interesting to note that total locomotion displayed by drug-free animals in the CS+ compartment was not correlated to the effect of post-training exposure to this CS+ on object memory, possibly suggesting that the two conditioned responses are dissociable. In addition, Experiment 5 with nicotine, and previous place conditioning studies with cocaine (see Introduction), demonstrated that a contextual CS+ established using the same protocol and the same apparatus, also acquires the ability to elicit conditioned approach (Fig. 6B). Unfortunately, place preference precludes an investigation of the correlation between conditioned approach and memory modulation because the test of preference involves a choice between the simultaneous presentation of the CS+ and the CS−. Hence, other conditioning preparations are needed to explicitly explore the relationship between conditioned locomotion, conditioned approach, and conditioned memory modulation in the same animals (Ettenberg 2009; Saunders et al. 2018).

The secondary finding of this study is that OR was no longer facilitated when exposure to the cocaine CS+ (Fig. 5B) or nicotine CS+ (Fig. 5D) was delayed by 6 h following the sample phase. These results are essential to the interpretation of the data for three reasons. First, they rule out the possibility that OR was facilitated by some drug-induced nonspecific effects on perceptual, cognitive, or motor functions resulting from repeated administration during the conditioning period. Second, they rule out possible nonspecific effects of exposure to the CS+ on general perceptual, cognitive, or motor functioning. Last, the findings exclude possible nonspecific effects linked to arousal or stress caused by confinement in the conditioning compartments.

The parallel findings with cocaine and nicotine suggest that these drugs may modulate memory consolidation by activating overlapping neurochemical systems. One of these systems may be the basolateral amygdala (Roozendaal et al. 1999, 2006; McGaugh and Roozendaal 2002; Stern and Alberini 2013), as it is known that its ablation blocks memory enhancement produced by systemic cocaine (Cestari et al. 1996) and that bilateral intra-amygdala infusions of nicotine enhance memory (Barros et al. 2005). There is also evidence that the mesolimbic dopamine system may be involved. For example, the ventral tegmental area is a primary source of dopamine afferents to the basolateral amygdala, hippocampus, and prefrontal cortex (Beninger 1983; Schultz et al. 1997; Wassum and Izquierdo 2015), all areas involved in memory formation (White and McDonald 2002; Paré 2003; Browning et al. 2005); systemic and central modulation of dopamine activity modulates consolidation of fear memory (Castellano et al. 1991; Rossato et al. 2009; de Lima et al. 2011), as well as consolidation of OR memory (Rossato et al. 2013), and intraventral tegmental infusions of nicotine enhance consolidation of fear memory (Lima et al. 2013). Central cholinergic systems could also play a role (Vnek et al. 1996) as injections of both cocaine and nicotine increase cholinergic activity in the hippocampus (Mitchell et al. 1989; Toide and Arima 1989; Brazell et al. 1991; Imperato et al. 1993), and intrahippocampal infusions of nicotine enhance memory (Sharifzadeh et al. 2005).

The interesting possibility raised by the current results is that pathways of memory enhancement shared by acute cocaine and nicotine may also be involved in memory enhancement induced by exposure to their CS. In support of this hypothesis, there is evidence that the basolateral amygdala is required for the facilitation of memory consolidation induced by conditioned emotional stimuli (Holahan and White 2002, 2004; Goode et al. 2016). Moreover, this region has efferent projections to the perirhinal cortex (Pikkarainen and Pitkänen 2001), which is required for OR memory (Winters et al. 2004). Mesolimbic dopamine may also play a role as it modulates conditioned responses to Pavlovian stimuli (Parkinson et al. 2002; Darvas et al. 2014), provides a functional reward signal that drives conditioning (Kim et al. 2012), and modulation of dopamine receptors in the perirhinal cortex significantly influences long-term object memory (Balderas et al. 2013). Finally, cholinergic mechanisms mediate conditioned reinforcement elicited by both drug and natural reward-associated stimuli (Löf et al. 2007; Wickham et al. 2015) and activation of nicotinic receptors in the perirhinal cortex facilitate object memory (Melichercik et al. 2012).

In conclusion, consistent with the memory consolidation hypothesis of drugs of abuse (White 1996), the present results suggest that contextual stimuli paired with the effects of cocaine and nicotine enhance memory consolidation. These data in rats identify a psychological function of cocaine- and nicotine-associated stimuli that is likely to impact the development and maintenance of addictive behaviors.

## Materials and Methods

### Subjects

A total of 108 male Sprague–Dawley rats (Charles River, Quebec, Canada), weighing between 225 and 250 g at the beginning of the experiments were individually housed in standard rat cages (polycarbonate; 50.5 × 48.5 × 20 cm) with standard bedding and environmental enrichment, and were maintained on a reverse light–dark schedule (lights off at 07:00; on at 19:00). All testing was conducted during the dark period. Rats had access to 25 g per day of standard rat chow, and water was available ad libitum in home cages. All experiments were approved by the Animal Care Committee of the University of Guelph and were performed in accordance with recommendations provided by the Canadian Council on Animal Care.

### Apparatus

#### Locomotion and place conditioning

Six semitransparent Plexiglas chambers (University of Guelph, Guelph, ON, Canada) were used for place conditioning. Each chamber included two distinct compartments of equal size (30 × 40 × 26 cm) separated by a removable insert (dark gray PVC). A small square opening (10 × 10 cm) at the back of the insert allowed access to both compartments during habituation, conditioning, and test sessions, and an identical insert without an opening was used for conditioning. Distinct visual (marbled white and black pattern on the wall of one compartment and vertical white and black stripes on the wall of the other; objects external to the boxes including cabinets, tables, and computer) and tactile (one compartment in each chamber contained a black ceramic floor tile) cues were maintained constant throughout the experiment. Black wire mesh covered the front of each compartment allowing for automatic video tracking (EthoVision v3, Noldus, The Netherlands). The software was also used to create a virtual transition zone (approximately the size of a 400 g rat) creating a third, middle compartment. Time spent in this virtual compartment was not included in data analysis.

#### Object recognition

OR was tested using a Y-apparatus, which consisted of three arms of equal size (40 × 27 × 10 cm) constructed from solid white Plexiglas to prevent the rat from looking out into the room. One arm was designated as the start arm and contained a guillotine door 18 cm from the rear of the arm to confine the rat at the start of a trial. The remaining two arms served as choice arms. The objects used were copies made from plastic, ceramic, and glass. Objects ranged in height from 10 to 20 cm and varied with respect to their visual and tactile qualities. Objects were fixed to the floor using odorless reusable adhesive putty. Objects were always wiped with 50% ethanol before being placed into the apparatus to control for any olfactory cues that may influence exploration. A JVC Everio digital camera was mounted on a tripod above the apparatus to record all trials.

### Procedures

#### Experiment 1

Experiment 1 was designed to assess the effect of acute post-sample cocaine administration on OR memory. Thirty-six rats were habituated to the empty Y-apparatus for 5 min on two consecutive days before the beginning of testing. The test trials began 24 h after the second habituation session. Each trial consisted of two phases: a sample phase and a choice phase, separated by a 72 h retention interval. This retention interval was chosen as a “suboptimal” condition in which drug-naïve rats do not typically express memory (Melichercik et al. 2012). Rats were always exposed to new, never-before-seen objects on each trial.

During the sample phase, two identical novel objects were placed into the Y-apparatus at the end of each exploration arm. Each rat was placed in the start box, and the guillotine door was opened. Rats were allotted a maximum of 180 sec to explore objects or were removed if 25 sec of total object exploration was achieved, whichever came first. Object exploration was defined as directing the nose to the object at <2 cm and/or touching the object with the nose. Twenty-four rats were injected immediately after the conclusion of the sample phase with 0, 5, 10, or 20 mg/kg cocaine. All animals were tested at each dose of cocaine and the order of cocaine doses was counterbalanced using a Latin Square Design. An additional group of 12 rats received 20 mg/kg cocaine 6 h following the conclusion of the sample phase. Following the 72 h retention interval, rats experienced the choice phase, for which the Y-apparatus contained a copy of the original sample object in one arm and a novel object in the other. The choice phase lasted 2 min, and the time spent exploring the novel and familiar objects was recorded. Different object pairs were used for each trial, and the order of exposure to object pairs, as well as the designated sample and novel objects for each trial were counterbalanced.

#### Experiment 2

Experiment 2 was designed to assess the effect of post-sample exposure to a cocaine (20 mg/kg)-conditioned context on OR memory. Forty-eight rats were habituated to each of the chambers for 30 min 24 h prior to the beginning of conditioning (vehicle in CS− and 20 mg/kg in CS+). At the beginning of conditioning, rats received either vehicle or 20 mg/kg cocaine and were immediately placed in the CS− or CS+ chamber for 2 h, respectively. The chambers of the apparatus used as CS− and CS+ were counterbalanced across rats. All animals received a total of 5 conditioning sessions with the CS− and 5 with the CS+, alternating over 10 successive days. Conditioned locomotion was assessed on two separate tests. The first test occurred the day after the last conditioning session and half of the animals were placed in the CS− and the other half were placed into the CS+. The second test occurred 72 h later and the same animals were tested in the alternate chamber.

Of the 48 rats, 24 rats were tested on OR and 24 rats were only tested on conditioning. The rats tested on OR were habituated to the Y-apparatus on Days 9 and 10 of conditioning and were exposed to the sample phase prior to the first test of conditioned locomotion on Day 11. Therefore, 12 of these subjects were exposed to the CS− immediately following exposure to the two objects, and the other 12 were exposed to the CS+ immediately following exposure to the two objects. The choice phase of OR occurred 72 h later (Day 14). On Day 15, the same animals experienced another sample phase of OR with different objects, and right after they were confined to the alternative conditioning chamber (CS− or CS+). The final test of OR occurred 72 h later (Day 18). Finally, this experiment also included a group of 12 rats that were tested as described above, but exposure to the CS− and CS+ was delayed by 6 h following the two sample phases.

#### Experiment 3

Experiment 3 was designed to assess the effect of acute post-sample nicotine administration on OR memory. The OR task was conducted using the same procedures as in Experiment 1, but rats received immediate post-sample (*n* = 24) 0, 0.1, 0.2, and 0.4 kg nicotine counterbalanced within subjects, or delayed (*n* = 12) 0.4 mg/kg nicotine.

#### Experiment 4

Experiment 4 was designed to assess the effect of post-sample exposure to a nicotine (0.4 mg/kg)-conditioned context on OR memory. A total of 48 rats were conditioned and tested on OR using the same procedures as in Experiment 2. Hence, 24 were only conditioned, 12 were also exposed to the nicotine-paired CS− and CS+ immediately after the sample phase, and 12 were exposed to the nicotine-paired CS− and CS+ 6 h following the sample phase.

#### Experiment 5

Experiment 5 was designed to assess the effect of nicotine (0.4 mg/kg) in place preference using an unbiased design. Nicotine has been shown to produce both conditioned place aversion and preference at various doses; therefore, we designed this experiment to assess the reinforcing effects of 0.4 mg/kg nicotine. Twelve rats were habituated for 30 min to the conditioning chambers 24 h prior to the beginning of conditioning (nicotine-paired chamber; vehicle-paired chamber). At the beginning of conditioning, rats received either vehicle or 0.4 mg/kg nicotine and were immediately placed in the vehicle-to-be paired chamber or nicotine-to-be paired chamber for 2 h. The chambers of the apparatus were counterbalanced across rats. All animals received a total of five conditioning sessions with vehicle and 5 with 0.4 mg/kg nicotine, alternating over 10 successive days. Place preference was assessed 24 h following the final conditioning day.

### Drugs

All drugs were injected intraperitoneally (i.p.). Vehicle (sterile 0.9% saline) was administered at 1 mL/kg. Cocaine hydrochloride at 5, 10, or 20 mg/kg (Dumex, Toronto, ON, Canada) and (−)Nicotine hydrogen tartrate salt at 0.1, 0.2, and 0.4 mg/kg (Sigma) was dissolved in sterile 0.9% physiological saline. The doses of these two drugs were selected because of their known stimulatory properties (Zavala et al. 2008) and their faciliatory effects on OR memory (Melichercik et al. 2012; Rkieh et al. 2014).

### Data analysis

One-, two-, and three-factor repeated measures analyses of variance (ANOVAs) were used as appropriate. Significant main effects, and/or interactions, were further analyzed by Student–Newman–Keuls post-hoc analysis. The one- and two-factor ANOVAs were performed using SigmaStat (v.3.5 for Windows). Three-factor ANOVAs were performed using GB-STAT, and the α level was ≤0.05. A discrimination ratio was used as a primary measure of OR and was calculated as (time exploring the novel object − time exploring the familiar object)/total time spent exploring both objects. Comparison between the sample and choice phase discrimination ratios was used as an index of successful memory for objects. Because the sample objects are identical, the sample phase discrimination ratio should be approximately 0; as such, a significant difference between choice and sample discrimination ratios is indicative of successful novelty/familiarity discrimination. For all OR experiments, total object exploration was also analyzed for both the sample and choice phases as a control measure of general exploratory behavior. The values of nonsignificant analyses are not reported. One rat from Experiment 1 and one from Experiment 3 had to be removed from data analysis because of complete inactivity during OR testing. One rat from Experiment 5 had to be removed because its habituation activity was two SDs above the mean.

## References

[LM048579WOLC1] Ahrens AM, Ma ST, Maier EY, Duvauchelle CL, Schallert T. 2009 Repeated intravenous amphetamine exposure: rapid and persistent sensitization of 50-kHz ultrasonic trill calls in rats. Behav Brain Res 197: 205–209. 10.1016/j.bbr.2008.08.037 18809437 PMC3969445

[LM048579WOLC18] Balderas I, Moreno-Castilla P, Bermudez-Rattoni F. 2013 Dopamine D1 receptor activity modulates object recognition memory consolidation in the perirhinal cortex but not in the hippocampus. Hippocampus 23: 873–878. 23674387 10.1002/hipo.22143

[LM048579WOLC2] Bardo MT, Rowlett JK, Harris MJ. 1995 Conditioned place preference using opiate and stimulant drugs: a meta-analysis. Neurosci Biobehav Rev 19: 39–51. 10.1016/0149-7634(94)00021-R 7770196

[LM048579WOLC3] Barros DM, Ramirez MR, Izquierdo I. 2005 Modulation of working, short- and long-term memory by nicotinic receptors in the basolateral amygdala in rats. Neurobiol Learn Mem 83: 113–118. 10.1016/j.nlm.2004.10.001 15721794

[LM048579WOLC4] Beer AL, Vartak D, Greenlee MW. 2013 Nicotine facilitates memory consolidation in perceptual learning. Neuropharmacology 64: 443–451. 10.1016/j.neuropharm.2012.06.019 22749926

[LM048579WOLC5] Beninger RJ. 1983 The role of dopamine in locomotor activity and learning. Brain Res Rev 6: 173–196. 10.1016/0165-0173(83)90038-3 6357357

[LM048579WOLC6] Berrettini W. 2008 Nicotine addiction. Am J Psychiatry 165: 1089–1092. 10.1176/appi.ajp.2008.08050780 18765487

[LM048579WOLC7] Blaiss CA, Janak PH. 2006 Post-training and post-reactivation administration of amphetamine enhances morphine conditioned place preference. Behav Brain Res 171: 329–337. 10.1016/j.bbr.2006.04.018 16698095 PMC1592232

[LM048579WOLC8] Blanco E, Bilbao A, Luque-Rojas MJ, Palomino A, Bermúdez-Silva FJ, Suárez J, Santín LJ, Estivill-Torrús G, Gutiérrez A, Campos-Sandoval JÁ, 2012 Attenuation of cocaine-induced conditioned locomotion is associated with altered expression of hippocampal glutamate receptors in mice lacking LPA1 receptors. Psychopharmacology (Berl) 220: 27–42. 10.1007/s00213-011-2446-6 21887497

[LM048579WOLC9] Bloch S, Bakay Pragay E, Mirsky AF. 1973 Heart rate and respiratory rate changes during drug-induced impairment in a conditioned avoidance task in monkeys. Pharmacol Biochem Behav 1: 29–34. 10.1016/0091-3057(73)90051-8 4204512

[LM048579WOLC10] Brazell MP, Mitchell SN, Gray JA. 1991 Effect of acute administration of nicotine on in vivo release of noradrenaline in the hippocampus of freely moving rats: a dose-response and antagonist study. Neuropharmacology 30: 823–833. 10.1016/0028-3908(91)90116-S 1685769

[LM048579WOLC11] Browning PG, Easton A, Buckley MJ, Gaffan D. 2005 The role of prefrontal cortex in object-in-place learning in monkeys. Eur J Neurosci 22: 3281–3291. 10.1111/j.1460-9568.2005.04477.x 16367793

[LM048579WOLC12] Calcagnetti DJ, Keck BJ, Quatrella LA, Schechter MD. 1995 Blockade of cocaine-induced conditioned place preference: relevance to cocaine abuse therapeutics. Life Sci 56: 475–483. 10.1016/0024-3205(94)00414-N 7869827

[LM048579WOLC13] Carrera MRA, Meijler MM, Janda KD. 2004 Cocaine pharmacology and current pharmacotherapies for its abuse. Bioorg Med Chem 12: 5019–5030. 10.1016/j.bmc.2004.06.018 15351386

[LM048579WOLC14] Castellano C, Cestari V, Cabib S, Puglisi-Allegra S. 1991 Post-training dopamine receptor agonists and antagonists affect memory storage in mice irrespective of their selectivity for D1 or D2 receptors. Behav Neural Biol 56: 283–291. 10.1016/0163-1047(91)90439-W 1684703

[LM048579WOLC15] Cestari V, Mele A, Oliverio A, Castellano C. 1996 Amygdala lesions block the effect of cocaine on memory in mice. Brain Res 713: 286–289. 10.1016/0006-8993(95)01556-6 8725002

[LM048579WOLC16] Ciamei A, Cestari V, Castellano C. 2000 Strain-dependent interactions between MK-801 and cocaine on retention of C57BL/6 and DBA/2 mice tested in a one-trial inhibitory avoidance task: involvement of dopaminergic mechanisms. Neurobiol Learn Mem 73: 188–194. 10.1006/nlme.1999.3932 10704328

[LM048579WOLC17] Ciamei A, Aversano M, Cestari V, Castellano C. 2001 Effects of MK-801 and nicotine combinations on memory consolidation in CD1 mice. Psychopharmacology (Berl) 154: 126–130. 10.1007/s002130000584 11314674

[LM048579WOLC19] Cummins Jacklin E, Boughner E, Kent K, Kwiatkowski D, MacDonald T, Leri F. 2015 Memory of a drug lapse: role of noradrenaline. Neuropharmacology 99: 98–105. 10.1016/j.neuropharm.2015.07.020 26192542

[LM048579WOLC20] Darvas M, Wunsch AM, Gibbs JT, Palmiter RD. 2014 Dopamine dependency for acquisition and performance of Pavlovian conditioned response. Proc Natl Acad Sci 111: 2764–2769. 10.1073/pnas.1400332111 24550305 PMC3932907

[LM048579WOLC21] Deiana S, Platt B, Riedel G. 2011 The cholinergic system and spatial learning. Behav Brain Res 221: 389–411. 10.1016/j.bbr.2010.11.036 21108971

[LM048579WOLC22] de Lima MNM, Presti-Torres J, Dornelles A, Siciliani Scalco FS, Roesler R, Garcia VA, Schröder N. 2011 Modulatory influence of dopamine receptors on consolidation of object recognition memory. Neurobiol Learn Mem 95: 305–310. 10.1016/j.nlm.2010.12.007 21187154

[LM048579WOLC23] Di Ciano P, Everitt BJ. 2003 Differential control over drug-seeking behavior by drug-associated conditioned reinforcers and discriminative stimuli predictive of drug availability. Behav Neurosci 117: 952–960. 10.1037/0735-7044.117.5.952 14570545

[LM048579WOLC24] Eddins D, Petro A, Williams P, Cerutti DT, Levin ED. 2009 Nicotine effects on learning in zebrafish: the role of dopaminergic systems. Psychopharmacology (Berl) 202: 103–109. 10.1007/s00213-008-1287-4 18716760

[LM048579WOLC25] Ennaceur A, Meliani K. 1988 A new one-trial test for neurobiological studies of memory in rats. III. Spatial vs. non-spatial working memory. Behav Brain Res 31: 47–59. 1482548 10.1016/s0166-4328(05)80315-8

[LM048579WOLC26] Ettenberg A. 2009 The runway model of drug self-administration Aaron. Pharmacol Biochem Behav 91: 271–277. 10.1016/j.pbb.2008.11.003 19032964 PMC2635890

[LM048579WOLC27] Fitzgerald RD, Francisco DL, Metcalfe J, Lawson MS. 1984 Classically conditioned heart rate and respiratory-motor activity in newborn and neonatal pygmy goats. Anim Learn Behav 12: 217–222.

[LM048579WOLC28] Goode TD, Leong KC, Goodman J, Maren S, Packard MG. 2016 Enhancement of striatum-dependent memory by conditioned fear is mediated by β-adrenergic receptors in the basolateral amygdala. Neurobiol Stress 3: 74–82. 10.1016/j.ynstr.2016.02.004 27981180 PMC5146203

[LM048579WOLC29] Hamed A, Taracha E, Szyndler J, Krzaścik P, Lehner M, Maciejak P, Skórzewska A, Płaźnik A. 2012 The effects of morphine and morphine conditioned context on 50kHz ultrasonic vocalisation in rats. Behav Brain Res 229: 447–450. 10.1016/j.bbr.2012.01.053 22326697

[LM048579WOLC30] Holahan MR, White NM. 2002 Conditioned memory modulation, freezing, and avoidance as measures of amygdala-mediated conditioned fear. Neurobiol Learn Mem 275: 250–275. 10.1006/nlme.2001.4012 11848722

[LM048579WOLC31] Holahan MR, White NM. 2004 Amygdala inactivation blocks expression of conditioned memory modulation and the promotion of avoidance and freezing. Behav Neurosci 118: 24–35. 10.1037/0735-7044.118.1.24 14979780

[LM048579WOLC32] Holahan MR, White NM. 2013 Memory enhancement produced by post-training exposure to sucrose-conditioned cues. F1000Res 2: 22 10.12688/f1000research.2-22.v1 24358865 PMC3790601

[LM048579WOLC33] Huston JP, Mondadori C, Waser P. 1974 Facilitation of learning by reward of post-trial memory processes. Experientia 30: 1038–1040. 10.1007/BF01938996

[LM048579WOLC34] Huston JP, Mueller CC, Mondadori C. 1977 Memory facilitation by posttrial hypothalamic stimulation and other reinforcers: a central theory of reinforcement. Biobehav Rev 1: 143–150. 10.1016/0147-7552(77)90003-1

[LM048579WOLC35] Imperato A, Obinu MC, Gessa GL. 1993 Effects of cocaine and amphetamine on acetylcholine release in the hippocampus and caudate nucleus. Eur J Pharmacol 238: 377–381. 10.1016/0014-2999(93)90869-J 8405105

[LM048579WOLC36] Iñiguez SD, Charntikov S, Baella SA, Herbert MS, Bolaños-Guzmán CA, Crawford CA. 2012 Post-training cocaine exposure facilitates spatial memory consolidation in C57BL/6 mice. Hippocampus 22: 802–813. 10.1002/hipo.20941 21542053 PMC3154999

[LM048579WOLC37] Introini-Collison IB, McGaugh JL. 1989 Cocaine enhances memory storage in mice. Psychopharmacology (Berl) 99: 537–541. 10.1007/BF00589905 2594920

[LM048579WOLC38] Jackson KJ, Kota DH, Martin BR, Damaj MI. 2009 The role of various nicotinic receptor subunits and factors influencing nicotine conditioned place aversion. Neuropharmacology 56: 970–974. 10.1016/j.neuropharm.2009.01.023 19371584 PMC3821837

[LM048579WOLC39] Janak PH, Keppel G, Martinez JL. 1992 Cocaine enhances retention of avoidance conditioning in rats. Psychopharmacology (Berl) 106: 383–387. 10.1007/BF02245422 1570387

[LM048579WOLC40] Johnson SA, Sediqzadah S, Erb S. 2012 Expression and resilience of a cocaine-conditioned locomotor response after brief and extended drug-free periods. Behav Brain Res 230: 69–77. 10.1016/j.bbr.2012.01.049 22326371

[LM048579WOLC41] Kim KM, Baratta MV, Yang A, Lee D, Boyden ES, Fiorillo CD. 2012 Optogenetic mimicry of the transient activation of dopamine neurons by natural reward is sufficient for operant reinforcement. PLoS One 7: e33612 10.1371/journal.pone.0033612 22506004 PMC3323614

[LM048579WOLC42] Le Foll B, Goldberg SR. 2005 Nicotine induces conditioned place preferences over a large range of doses in rats. Psychopharmacology (Berl) 178: 481–492. 10.1007/s00213-004-2021-5 15765262

[LM048579WOLC43] Le Foll B, Goldberg SR. 2006 Nicotine as a typical drug of abuse in experimental animals and humans. Psychopharmacology (Berl) 184: 367–381. 10.1007/s00213-005-0155-8 16205918

[LM048579WOLC44] Le Foll B, Goldberg SR. 2009 Effects of nicotine in experimental animals and humans: an update on addictive properties. Handb Exp Pharmacol 192: 335–367. 10.1007/978-3-540-69248-5_12 PMC2687081 19184655

[LM048579WOLC45] Leong KC, Goodman J, Packard MG. 2015 Post-training re-exposure to fear conditioned stimuli enhances memory consolidation and biases rats toward the use of dorsolateral striatum-dependent response learning. Behav Brain Res 291: 195–200. 10.1016/j.bbr.2015.05.022 26005126

[LM048579WOLC46] Leri F, Zhou Y, Goddard B, Cummins E, Kreek MJ. 2006 Effects of high-dose methadone maintenance on cocaine place conditioning, cocaine self-administration, and μ-opioid receptor mRNA expression in the rat brain. Neuropsychopharmacology 31: 1462–1474. 10.1038/sj.npp.1300927 16237390

[LM048579WOLC47] Leri F, Nahas E, Henderson K, Limebeer CL, Parker LA, White NM. 2013 Effects of post-training heroin and d-amphetamine on consolidation of win-stay learning and fear conditioning. J Psychopharmacol 27: 292–301. 10.1177/0269881112472566 23325371

[LM048579WOLC048] Lima RH, Radiske A, Köhler CA, Gonzalez MC, Bevilaqua LR, Rossato JI, Medina JH, Cammarota M. 2013 Nicotine modulates the long-lasting storage of fear memory. Learn Mem 20: 120–124. 23418390 10.1101/lm.029900.112

[LM048579WOLC48] Liu Y, Le Foll B, Liu Y, Wang X, Lu L. 2008 Conditioned place preference induced by licit drugs: establishment, extinction, and reinstatement. ScientificWorldJournal 8: 1228–1245. 10.1100/tsw.2008.154 19082419 PMC5848638

[LM048579WOLC49] Löf E, Olausson P, deBejczy A, Stomberg R, McIntosh JM, Taylor JR, Söderpalm B. 2007 Nicotinic acetylcholine receptors in the ventral tegmental area mediate the dopamine activating and reinforcing properties of ethanol cues. Psychopharmacology (Berl) 195: 333–343. 10.1007/s00213-007-0899-4 17703283

[LM048579WOLC50] Ma ST, Maier EY, Ahrens AM, Schallert T, Duvauchelle CL. 2010 Repeated intravenous cocaine experience: development and escalation of pre-drug anticipatory 50-kHz ultrasonic vocalizations in rats. Behav Brain Res 212: 109–114. 10.1016/j.bbr.2010.04.001 20382187 PMC2873056

[LM048579WOLC51] May Z, Morrill A, Holcombe A, Johnston T, Gallup J, Fouad K, Schalomon M, Hamilton TJ. 2016 Object recognition memory in zebrafish. Behav Brain Res 296: 199–210. 10.1016/j.bbr.2015.09.016 26376244

[LM048579WOLC52] McGaugh JL. 2000 Memory – a century of consolidation. Science 287: 248–251. 10.1126/science.287.5451.248 10634773

[LM048579WOLC53] McGaugh JL, Roozendaal B. 2002 Role of adrenal stress hormones in forming lasting memories in the brain. Curr Opin Neurobiol 12: 205–210. 10.1016/S0959-4388(02)00306-9 12015238

[LM048579WOLC54] McGaugh JL, Roozendaal B. 2009 Drug enhancement of memory consolidation: historical perspective and neurobiological implications. Psychopharmacology (Berl) 202: 3–14. 10.1007/s00213-008-1285-6 18704369

[LM048579WOLC55] Melichercik AM, Elliott KS, Bianchi C, Ernst SM, Winters BD. 2012 Nicotinic receptor activation in perirhinal cortex and hippocampus enhances object memory in rats. Neuropharmacology 62: 2096–2105. 10.1016/j.neuropharm.2012.01.008 22280876

[LM048579WOLC56] Messier C, White NM. 1984 Contingent and non-contingent actions of sucrose and saccharin reinforcers: effects on taste preference and memory. Physiol Behav 32: 195–203. 10.1016/0031-9384(84)90129-X 6718546

[LM048579WOLC57] Mitchell SN, Brazell MP, Joseph MH, Alavijeh MS, Gray JA. 1989 Regionally specific effects of acute and chronic nicotine on rates of catecholamine and 5-hydroxytryptamine synthesis in rat brain. Eur J Pharmacol 167: 311–322. 10.1016/0014-2999(89)90440-8 2509220

[LM048579WOLC58] Paré D. 2003 Role of the basolateral amygdala in memory consolidation. Prog Neurobiol 70: 409–420. 10.1016/S0301-0082(03)00104-7 14511699

[LM048579WOLC59] Parkinson JA, Dalley JW, Cardinal RN, Bamford A, Fehnert B, Lachenal G, Rudarakanchana N, Halkerston KM, Robbins TW Everitt BJ. 2002 Nucleus accumbens dopamine depletion impairs both acquisition and performance of appetitive Pavlovian approach behaviour: implications for mesoaccumbens dopamine function. Behav Brain Res 137: 149–163. 10.1016/S0166-4328(02)00291-7 12445721

[LM048579WOLC60] Pikkarainen M, Pitkänen A. 2001 Projections from the lateral, basal and accessory basal nuclei of the amygdala to the perirhinal and postrhinal cortices in rat. Cereb Cortex 11: 1064–1082. 11590116 10.1093/cercor/11.11.1064

[LM048579WOLC61] Puglisi-Allegra S, Cestari V, Cabib S, Castellano C. 1994 Strain-dependent effects of post-training cocaine or nomifensine on memory storage involve both D1 and D2 dopamine receptors. Psychopharmacology (Berl) 115: 157–162. 10.1007/BF02244766 7862889

[LM048579WOLC62] Puma C, Deschaux O, Molimard R, Bizot JC. 1999 Nicotine improves memory in an object recognition task in rats. Eur Neuropsychopharmacol 9: 323–327. 10.1016/S0924-977X(99)00002-4 10422893

[LM048579WOLC63] Rescorla RA, Solomon RL. 1975 Two process learning theory: relationships between Pavlovian conditioning and instrumental learning. Psychol Rev 74: 151–182. 10.1037/h0024475 5342881

[LM048579WOLC64] Risinger FO, Oakes RA. 1995 Nicotine-induced conditioned place preference and conditioned place aversion in mice. Pharmacol Biochem Behav 51: 457–461. 10.1016/0091-3057(95)00007-J 7667368

[LM048579WOLC65] Rkieh N, Cloke JM, Gallagher N, Winters BD, Leri F. 2014 Drugs of abuse as memory modulators: a study of cocaine in rats. Psychopharmacology (Berl) 231: 2339–2348. 10.1007/s00213-013-3390-4 24337026

[LM048579WOLC66] Roozendaal B, McGaugh JL. 2012 Memory modulation. Behav Neurosci 125: 797–824. 10.1037/a0026187 PMC3236701 22122145

[LM048579WOLC67] Roozendaal B, Nguyen BT, Power AE, McGaugh JL. 1999 Basolateral amygdala noradrenergic influence enables enhancement of memory consolidation induced by hippocampal glucocorticoid receptor activation. Proc Natl Acad Sci 96: 11642–11647. 10.1073/pnas.96.20.11642 10500230 PMC18087

[LM048579WOLC68] Roozendaal B, Okuda S, Van der Zee EA, McGaugh JL. 2006 Glucocorticoid enhancement of memory requires arousal-induced noradrenergic activation in the basolateral amygdala. Proc Natl Acad Sci 103: 6741–6746. 10.1073/pnas.0601874103 16611726 PMC1458951

[LM048579WOLC69] Rossato JI, Bevilaqua LR, Izquierdo I, Medina JH, Cammarota M. 2009 Dopamine controls persistence of long-term memory storage. Science 325:1017–1020. 10.1126/science.1172545 19696353

[LM048579WOLC70] Rossato JI, Radiske A, Kohler CA, Gonzalez C, Bevilaqua LR, Medina JH, Cammarota M. 2013 Consolidation of object recognition memory requires simultaneous activation of dopamine D1/D5 receptors in the amygdala and medial prefrontal cortex but not in the hippocampus. Neurobiol Learn Mem 106: 66–70. 10.1016/j.nlm.2013.07.012 23891712

[LM048579WOLC71] Sansone M, Castellano C, Battaglia M, Ammassari-Teule M. 1991 Effects of oxiracetam-nicotine combinations on active and passive avoidance learning in mice. Pharmacol Biochem Behav 39: 197–200. 10.1016/0091-3057(91)90421-W 1924503

[LM048579WOLC72] Saunders BT, Richard JM, Margolis EB, Janak PH. 2018 Dopamine neurons create Pavlovian conditioned stimuli with circuit-defined motivational properties. Nat Neurosci 21: 1072–1083. 10.1038/s41593-018-0191-4 30038277 PMC6082399

[LM048579WOLC73] Schultz W, Dayan P, Montague PR. 1997 A neural substrate of prediction and reward. Science 275: 1593–1599. 10.1126/science.275.5306.1593 9054347

[LM048579WOLC74] Sharifzadeh M, Tavasoli M, Naghdi N, Ghanbari A, Amini M, Roghani A. 2005 Post-training intrahippocampal infusion of nicotine prevents spatial memory retention deficits induced by the cyclo-oxygenase-2-specific inhibitor celecoxib in rats. J Neurochem 95: 1078–1090. 10.1111/j.1471-4159.2005.03454.x 16150053

[LM048579WOLC75] Stern SA, Alberini CM. 2013 Mechanisms of memory enhancement. Wiley Interdiscip Rev Syst Biol Med 5: 37–53. 10.1002/wsbm.1196 23151999 PMC3527655

[LM048579WOLC76] Sticht M, Mitsubata J, Tucci M, Leri F. 2010 Reacquisition of heroin and cocaine place preference involves a memory consolidation process sensitive to systemic and intra-ventral tegmental area naloxone. Neurobiol Learn Mem 93: 248–260. 10.1016/j.nlm.2009.10.005 19857583

[LM048579WOLC77] Thériault RK, Leri F, Kalisch B. 2018 The role of neuronal nitric oxide synthase in cocaine place preference and μ opioid receptor expression in the nucleus accumbens. Psychopharmacology 235: 2675–2685. 10.1007/s00213-018-4961-1 29992335

[LM048579WOLC78] Thorndike EL. 1911 Animal intelligence. Macmillan, New York.

[LM048579WOLC79] Toide K, Arima T. 1989 Effects of cholinergic drugs on extracellular levels of acetylcholine and choline in rat cortex, hippocampus and striatum studied by brain dialysis. Eur J Pharmacol 173: 133–141. 10.1016/0014-2999(89)90510-4 2625133

[LM048579WOLC80] Tunstall BJ, Kearns DN. 2017 Cocaine can generate a stronger conditioned reinforcer than food despite being a weaker primary reinforcer. Addict Biol 21: 282–293. 10.1111/adb.12195 PMC4417659 25363637

[LM048579WOLC81] Tzschentke TM. 1998 Measuring reward with the conditioned place preference paradigm: a comprehensive review of drug effects, recent progress and new issues. Prog Neurobiol 56: 613–672. 10.1016/S0301-0082(98)00060-4 9871940

[LM048579WOLC82] Vnek N, Kromer LF, Wiley RG, Rothblat LA. 1996 The basal forebrain cholinergic system and object memory in the rat. Brain Res 710: 265–270. 10.1016/0006-8993(95)01477-2 8963668

[LM048579WOLC83] Wassum KM, Izquierdo A. 2015 The basolateral amygdala in reward learning and addiction. Neurosci Biobehav Rev 57: 271–283. 10.1016/j.neubiorev.2015.08.017 26341938 PMC4681295

[LM048579WOLC84] White NM. 1996 Addictive drugs as reinforcers: multiple partial actions on memory systems. Addiction 91: 921–949. 10.1111/j.1360-0443.1996.tb03586.x 8688822

[LM048579WOLC85] White NM, McDonald RJ. 2002 Multiple parallel memory systems in the brain of the rat. Neurobiol Learn Mem 77: 125–184. 10.1006/nlme.2001.4008 11848717

[LM048579WOLC86] White NM, Milner PM. 1992 The psychobiology of reinforcers. Annu Rev Psychol 43: 443–471. 10.1146/annurev.ps.43.020192.002303 1539948

[LM048579WOLC87] Wickham RJ, Solecki WB, Nunes EJ, Addy NA. 2015 Distinct effects of ventral tegmental area NMDA and acetylcholine receptor blockade on conditioned reinforcement produced by food-associated cues. Neuroscience 301: 384–394. 10.1016/j.neuroscience.2015.06.021 26093048 PMC4510872

[LM048579WOLC88] Winters BD, Forwood SE, Cowell RA, Saksida LM, Bussey TJ. 2004 Double dissociation between the effects of peri-postrhinal cortex and hippocampal lesions on tests of object recognition and spatial memory: heterogeneity of function within the temporal lobe. J Neurosci 24: 5901–5908. 10.1523/JNEUROSCI.1346-04.2004 15229237 PMC6729235

[LM048579WOLC89] Zavala AR, Browning JR, Dickey ED, Biswas S, Neisewander JL. 2008 Region-specific involvement of AMPA/Kainate receptors in Fos protein expression induced by cocaine-conditioned cues. Eur Neuropsychopharmacol 18: 600–611. 10.1016/j.euroneuro.2008.04.010 18539009 PMC4798851

[LM048579WOLC089] Zhai HF, Zhang ZY, Zhao M, Qiu Y, Ghitza UE, Lu L. 2007 Conditioned drug reward enhances subsequent spatial learning and memory in rats. Psychopharmacology 195: 193–201. 17661018 10.1007/s00213-007-0893-x

